# Lost on the Pacific Crest Trail: a 4,270 km survey of wilderness waste distribution and characteristics

**DOI:** 10.1016/j.wasman.2025.115063

**Published:** 2025-08-13

**Authors:** Victoria McGruer, Macy Gustavus, Kendra Z. Hess, Hazel Vaquero, Krystle Moody Wood, Emin Israfil, Jorge Gonzalez-Estrella, Victoria M. Fulfer, Shelly Moore, Win Cowger

**Affiliations:** a Moore Institute for Plastic Pollution Research, Long Beach, CA, USA; b Toxicology Department, Southern California Coastal Water Research Project, Costa Mesa, CA, USA; c School of Civil & Environmental Engineering, Oklahoma State University, Stillwater, OK, USA; d Materevolve, LLC, San Anselmo, CA, USA; e Rubbish, San Francisco, CA, USA; f The 5 Gyres Institute, Santa Monica, CA, USA; g University of California, Riverside, Riverside, CA, USA

**Keywords:** Wilderness, Waste, Plastic pollution, Recreation trails, Pacific Crest Trail, Land management

## Abstract

Mismanaged waste threatens the environment and human health. To better understand waste sources and impacts along recreation trails, we surveyed the entire 4,270 km Pacific Crest Trail, conducting 251 waste surveys (1 km every 16 km). Surveys counted and classified waste within 2 m of the trail. We estimated that there were 53,000 pieces (12,000–130,000) of waste along the trail in 2023, based on a mean count of 12.5 pieces per km. Waste count had a negative log–log linear relationship with distance to the nearest road. Far in the backcountry (survey start 10 km from a road), waste was sparse (0.4 pieces per km), but close to roads (10 m from a road), waste increased to 13 pieces per km. The most common material types were soft plastic (36 %), metal (11 %), and sanitary waste (10 %). The most common morphologies were fragments (47 %), package ends (7 %), and wipes (6 %). Brands visible on waste were rare (48 pieces). We assessed the bias of this survey method, showing that it underestimated waste counts by 50 % compared to conducting the survey twice or with twice the number of surveyors. Surveying trail sections with snow cover or at night also reduced observed counts. These findings suggest that cleanup near roads, waste handling education, reduced plastic use, and innovation from outdoor consumer product producers could reduce trail waste. We propose that a baseline value of 0.4 pieces per km should be a waste management target to achieve for all spaces.

## 1. Introduction

Mismanaged waste (e.g., plastic, sanitary products, tobacco products) is a major concern for the environment and human health (Thompson et al., 2024; Umar Donuma et al., 2024). There are clear impacts on terrestrial organisms from ingesting waste, which can cause intestinal blockage leading to death (Eriksen et al., 2021; Hailat et al., 1997; Meyer et al., 2023). Additionally, waste may leach hazardous chemicals (Coffin et al., 2018) and fragment with time into smaller pieces (Andrady, 2011), becoming a dominant source of anthropogenic microparticles (e.g., microplastics) with additional ecological consequences (Barnes et al., 2009). Waste can also present hazards to humans, such as biological illnesses (e.g., contaminated tissue paper), invasive species (e.g., food waste), or lacerations (e.g., broken glass or metal). The backcountry (> 10 km from roads) is an important system for preservation of animals and enjoyment of humans (Mittermeier et al., 2003) and waste should not be present, but it is. Wilderness waste is difficult to remediate due to access limitations, and materials like plastic can persist (Chamas et al., 2020) for hundreds of years, accumulating in the environment. Trails are one of the primary conduits that humans use to participate in the wilderness (Snead et al., 2011).

To date, few surveys have been conducted on trail waste to quantify abundance, type, and controls. An early study in 1989 (Cutter et al., 1991) found that waste along pathways in New Jersey state parks (6600 pieces per km) had 50 % less waste than urban areas and that cigarette waste was the most prevalent type. A more recent study (2009) of recreational trails in New Hampshire and Maine found 1–6 pieces per km, with motorized trails having 5 times more waste than non-motorized trails (Wilkerson and Whitman, 2009). In contrast, a survey of macroplastics on trails in the Italian Central Western Alps found that food packaging was the most prevalent, with 2–26 items per km, demonstrating the waste impact from mountaineering activities (Parolini et al., 2021). A long term study of trail waste in Poland found that mismanaged waste generated per hiker decreased by 30 % between 2012 and 2017 (Religa and Adach, 2020). These studies highlight the complexity of managing wilderness waste due to the diversity of waste abundance, type, sources, hotspots, and management opportunities, and underscore the need for continued scientific investigation.

The Pacific Crest Trail travels 4,270 km from the United States border with Mexico to Canada through California, Oregon, and Washington, crossing through 7 national parks, 24 national forests, and 48 congressionally designated wilderness areas. It is estimated that hundreds of thousands of people (“PCT visitor use statistics,” 2024) use the trail annually with most use happening during Spring, Summer, and Fall. Trails are an extension of the human transportation network into the wilderness, although distinct from roads by management and use (Snead et al., 2011). Some sections of the Pacific Crest Trail even walk along the side of a major road. California is leading the way for the country as one of the first states in the United States to define targets for low waste abundance in roadside environments (~100 pieces per road km) and mandate monitoring, mitigation, and reporting (CalTrans 2023 Trash Assessment and Implementation Plan, 2023). The 100 pieces per km threshold has emerged from over a decade of research to determine a baseline value for “low” waste conditions on roadsides that all roads in California must reach. It is bold and already having positive impacts on the environment. We asked the question, how close is this threshold to the cleanliness of a typical wilderness trail? Reaching that target would ensure that roads do not contribute to waste in the environment more than a typical wilderness trail would. The diversity in management, ecosystem, and visitor use makes the Pacific Crest Trail ideal for conducting generalizable research that applies to many wilderness management groups and policy applications.

The study aimed to assess the abundance and type of waste (> 1 cm) across the entire Pacific Crest Trail, identify waste sources, establish baseline conditions of waste, develop a new method for reproducibly measuring trail waste, and identify opportunities for improved wilderness waste management. We estimated the total waste along the trail and calculated the average concentration to establish a baseline and assess hotspots. Our primary hypothesis was that waste abundance correlated with the distance to the nearest road. If so, we planned to use that relationship to estimate average conditions near roads versus backcountry conditions. Additionally, we evaluated the percentage of each type of waste to identify priority pollutants that could be targeted for prevention efforts to reduce wilderness waste. A new method for surveying trail waste was validated which when repeated will be able to assess trends through time. We thoroughly evaluated the biases associated with the methods employed and provided recommendations for improving future methodologies. Lastly, we discussed the management opportunities apparent from the findings. The novelty of this study comes from the total distance covered (4270 km), number of waste surveys (251), and scope (deep wilderness > 10 mi from a road). The next most extensive study we could find covered 335 km and conducted 112 surveys, mostly on easily accessible motorized trails (Wilkerson and Whitman, 2009). This provided us with high statistical power to address the stated aims and enabled us to tackle regional to national scale questions about wilderness trail waste management.

## 2. Methods

### 2.1. Survey approach

**Fig. 1.**
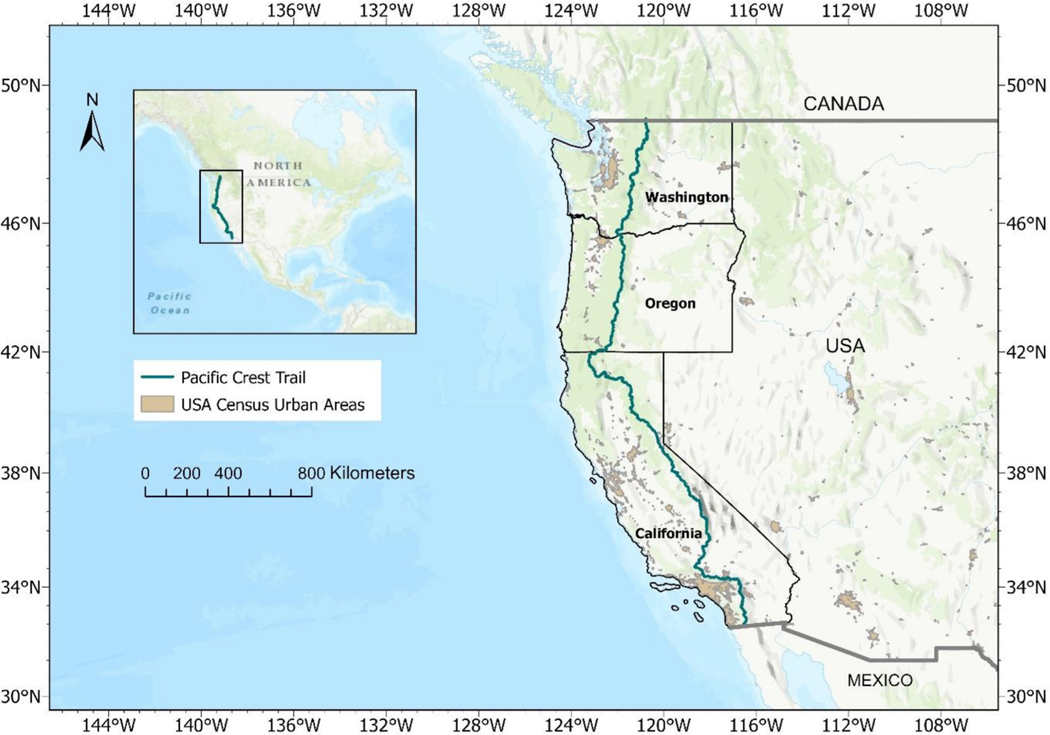
Map of the Pacific Crest Trail. Trail centerline in green stretches from Mexico to Canada through three of the United States states labeled on the map.

The study surveyed waste along the 4270 km Pacific Crest Trail from March to September 2023. A power analysis prior to the start of the study estimated that 204 surveys were required to obtain a 7 % uncertainty in waste concentration and characterization methods (Cowger et al., 2024a). The survey was designed to maximize data quality and richness, while minimizing survey time and effort to increase the likelihood of success in completing a long trail survey. The surveyors conducted waste surveys every 16 km (10 mi) on the Pacific Crest Trail (Fig. 1), resulting in a total of 251 unique surveys (1/16th of the entire trail length). Thirteen surveys were skipped due to trail closures or unsafe trail conditions. Surveys began at the US-Mexico border and extended to the US-Canada border. Sections of the trail through the California Sierra Nevada mountains from km 1132 (lat-lon = 36.024, −118.134) to 2144 (lat-lon = 40.261, −121.338) were initially skipped due to record-breaking snowpack. After completing the section from km 2144 to the US-Canada border, the surveyors finished the skipped section going north to south.

The start point of each survey was identified using the Far-Out app ([AuthorError] et al., 2024), which tracks GPS position relative to the trail distance. The Rubbish app (Rubbish, 2024a) and a Garmin watch were activated to record waste data and the 1 km distance surveyed, respectively. Rubbish is a cell phone application for collecting waste data. It allows for recording survey tracks, images, waste labels, time stamps, and geolocations. A georeferenced photo of the trail survey start was taken with a phone camera. The surveys were typically completed by one or two primary surveyors. To achieve the broader impact goal of engaging hikers about waste management, additional volunteers occasionally assisted with the surveys and were trained in the survey methodology. The number of surveyors was recorded in the notes of the Rubbish app for each survey to track potential surveyor number bias. Survey conditions were not always predictable, and to conduct as many surveys as possible safely, the surveyors occasionally had to survey at night and in snow-covered conditions (both conditions were expected to reduce waste counts). Night and snow surveys were labeled in the notes of the Rubbish app.

**Fig. 2.**
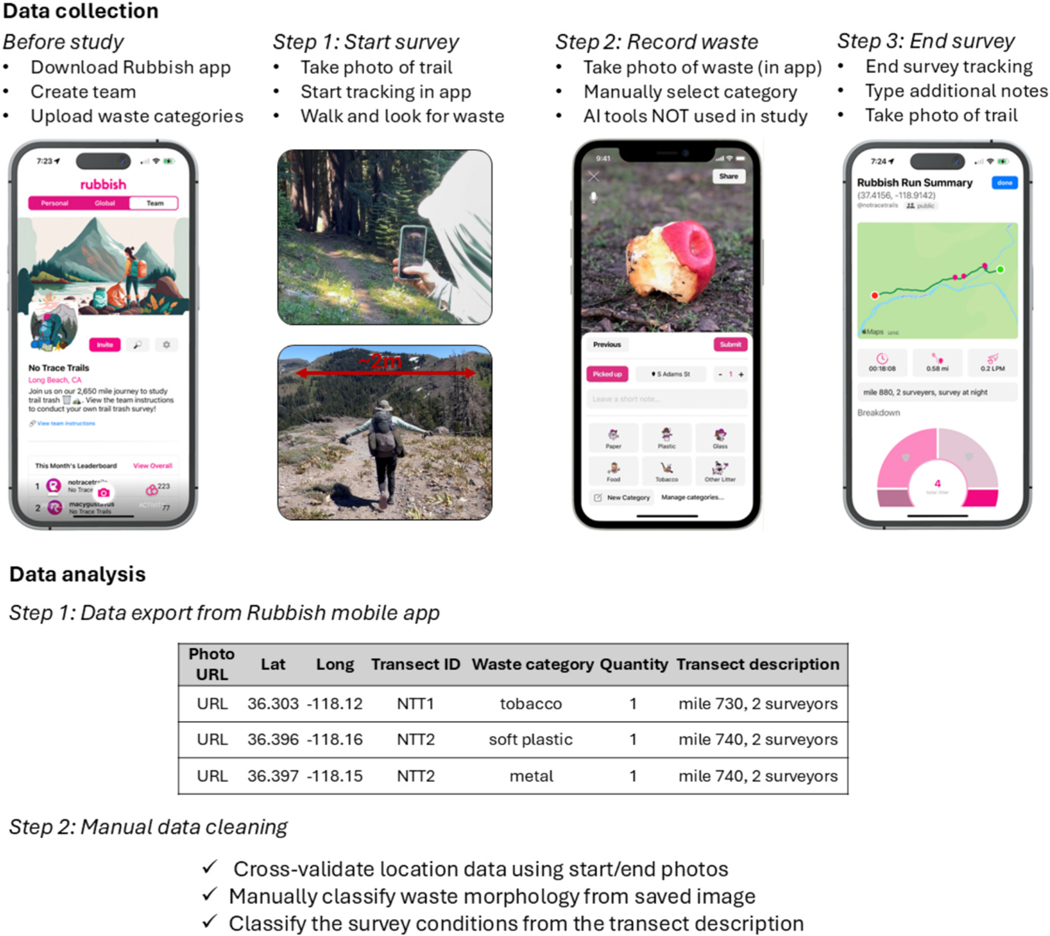
Data collection: Before the start of the study, the surveyors downloaded the Rubbish IOS application and created a team called “no trace trails” so that all surveys conducted by the team would be grouped in the app interface. A predetermined list of waste categories was uploaded to the app for use in categorizing items found. On trail, at the start of each survey (step 1), a georeferenced photo of the start of the survey was taken with a phone camera, and a GPS watch was used to track the distance of the transect (1 km). The surveyors then began walking down the trail, looking for waste on the trail or within approximately 2 m of the trail. When waste was encountered (step 2), a photo was taken using the Rubbish mobile app, and the waste was manually categorized according to the previously uploaded list. Note: The AI tools available in the Rubbish app were not used in this study. When the surveyors reached the end of the transect (step 3), the survey was ended in the Rubbish app, and the surveyors added additional notes for use in downstream data analysis (e.g., the number of surveyors, snow cover on the trail, whether the survey was conducted at night). **Data analysis:** Data collected during the survey was exported through the Rubbish data portal. The Rubbish application did not process the images or data outside of recording the surveyor’s direct input and location tracking. Data locations were cross-validated using georeferenced start and end photos collected throughout the survey. All data were checked by the study authors. Exported images of the waste were assessed by the authors, and waste morphology was manually categorized. Notes added at the end of each transect were used to categorize the survey conditions.

During the survey, surveyors would walk in the center of the trail, one behind the other. Any waste observed on the trail or within approximately 2 m (hiking poles out with arms extended) of either side of the trail was recorded. Using the Rubbish app, every piece of waste was recorded by taking an image of the item and labeling it by manually selecting the material type from a list of categories that were uploaded to the app ahead of the study. The eighteen material type categories recorded in the app were: glass, ceramic, plastic foam, plastic rubber, soft plastic, hard plastic, sanitary waste, feces, leather, food, wood, paper, cardboard, cloth, metal, tobacco-related, concrete or asphalt, and other litter. At the time of writing, image-based AI classification is also available in Rubbish, but this functionality was not available at the time of the study and was not used. Voice recognition to command the app was trialed in 2 surveys but did not provide improved time efficiency over manual entry because few pieces of waste were found per survey and some of the voice automated entries had to be reentered manually. For each image taken, Rubbish kept track of geolocation and timestamp using the phone’s readings, and merged the data across the two surveyors’ devices. Waste was collected and removed whenever possible. Each survey ended after a 1 km distance was covered (as measured by the Garmin watch) or 100 pieces were logged, whichever came first. The 100-piece limit was set to ensure that high-quality data was collected for all items by avoiding survey fatigue, and to prevent safety issues that could arise from unexpectedly staying in a remote area for too long without sufficient supplies. Recording 100 pieces takes approximately 1 h for a team of 2 and results in a 10 % uncertainty in waste type and abundance for the individual site, based on power analysis. To complete the survey, a georeferenced photo was taken of the end of the trail and the surveyors added additional notes within the Rubbish app describing the trail conditions (e.g., the number of surveyors, snow cover on the trail, whether the survey was conducted at night) (Fig. 2). Although a specific application (Rubbish) was used to record data during the surveys, there is no reason why a different waste recording application or approach could not record comparable data, so long as the field team has access to the date and time, their location relative to the trail distance, a camera to take photos of the waste, and a way to record observed waste types and counts.

Eight surveys were repeated (trail kilometers: 640, 960, 1440, 1904, 2400, 2912, 3360, 3840) immediately after survey completion, with the same number of surveyors, to assess the amount of waste that may have been missed during the first survey. Because these surveys required an additional 2 km of hiking in the opposite direction, repeat surveys were minimized to conserve resources. A detailed standard operating procedure and video explaining the data collection method are available in the supplemental information.

### 2.2. Analysis

#### 2.2.1. Data cleanup

All data were aggregated from the Rubbish application programming interface (API), a data portal (Rubbish, 2024b). The API provided images, waste categories assigned by the surveyors, survey location, timestamp, saved notes, and other data that we did not leverage in this study. Survey locations were georeferenced to the nearest mile marker from the Pacific Crest Trail Association dataset, which provides geospatial points of each mile marker location (Data and [WWW Document], 2024). Quality control surveys, where surveyors reassessed the same stretch of trail, were labeled as such and used only for calculating the single-survey bias. Data were quality-checked by the study authors. Images and survey locations were visually inspected to verify that the waste characteristics described by the surveyors were accurate and not double-counted. Records that the trail crew confirmed as inaccurate were corrected or removed from the dataset.

Data were standardized to prepare for statistical analysis, which assessed waste abundance and type. Waste categories were standardized to closely follow the categorization recommendations in the Trash Taxonomy (Hapich et al., 2022, 2024). The Trash Taxonomy is a categorization framework developed to enhance comparability and interpretability between waste surveys. Waste material types (described in 2.1) were the only characteristics labeled in the field, while waste morphology and brand were extracted from the images. Determining the material type often requires handling the object, whereas morphology and brand can be determined from the images. For sites where the surveyors reached 100 pieces before the end of the 1 km survey (n = 2), we extrapolated the counts to the full survey length by multiplying the observed count by the ratio between 1 km and the distance surveyed before reaching the 100th piece. Waste was not detected in 35 % of surveys. These zero counts can be highly problematic for regression and other statistical tests, producing inaccurate results (e.g., zero values cannot be log-transformed). It is considered best practice to replace these values with realistic values between zero and the limit of detection (here a count of 1)(Eriksen et al., 2023; Helsel, 2006). We corrected the zero values for abundance assessment only (not waste type) by using a regression on ordered statistics (cenros) model (Lee, 2020) to predict count values between one and zero. The model assumed the true underlying data came from a log-normal distribution and used the distribution of the data above one to predict the zero values. Waste abundance was estimated by calculating the mean count observed and multiplying it by the entire length of the trail. Confidence intervals (95 %) for all mean estimates were estimated using bootstrapping with replacement (n = 10,000) of the mean values.

#### 2.2.2. Result Calculations

Waste abundance was calculated as the total waste observed in each survey divided by the survey distance. The relationship between distance to roads and trail waste abundance was assessed by taking the beginning mile marker of each survey and buffering it by 36 1.25-factor increases: from 9 m up to 22,959 m. The buffers were then searched to determine if a road occurred within the buffer in Open Street Map (OpenStreetMap contributors, 2017), and the smallest buffer was assigned the distance to nearest road value for that point. A log–log linear regression was used to assess the relationship.

The most common waste materials and morphologies were assessed by calculating the mean percentage across all surveys. To do this, we first calculated the percentage of each type during each survey and then averaged (mean) across the surveys. Confidence intervals (95 %) for all mean estimates were estimated using bootstrapping with replacement (n = 10,000) of the mean values.

There were several survey conditions that we thought could bias the calculation of the mean waste count. Rather than excluding these surveys, these conditions were recorded and then assessed for their effect on the mean waste counts. The conditions evaluated included the effect of surveying at night by headlamp (n = 4; 2 % of surveys), the effect of surveying snow-covered trails (n = 22; 9 % of surveys), and the effect of having different numbers of surveyors (20 surveys had > 2 surveyors; 8 % of surveys). Additionally, we evaluated data from the eight surveys (3 % of surveys) that were repeated to estimate the amount of waste that may have been missed in the first survey. We estimated the bias of the night and snow effects by dividing the mean waste counts observed during surveys under these conditions by the mean waste counts observed during all other surveys. The resurvey bias was assessed by dividing the mean count observed on the first survey by the mean count observed on the second survey. The effect of the number of surveyors was assessed by fitting a linear regression between the waste count per km and the number of participating surveyors. One survey, which involved 5 people, was excluded from this specific analysis due to its outsized influence on the relationship, as the observed waste count in the survey was zero. Twelve surveys did not report a value for the number of surveyors. These surveys were excluded from the regression analysis but were used for all other analyses, including waste count estimation purposes. Missing surveyor numbers were replaced with the mean number of surveyors for the waste count estimation. This regression was then used to predict the mean waste count if all surveys had been completed with four surveyors. The predicted mean was then compared with the raw mean. We did not intend to correct for these biases in the final estimates, as this research is still in development; however, we assessed how a theoretical correction might impact the observed mean counts.

Statistics were calculated in R (R Core Team, 2020) (version 4.3.3) using packages jsonlite (Ooms, 2014), data.table (Dowle and Srinivasan, 2020), dplyr (Wickham et al., 2020), sf (Pebesma, 2018), tidyverse (Wickham et al., 2019), mapview (Appelhans et al., 2022), mapdata (Becker, 2022), RcolorBrewer (Neuwirth, 2022), httr (Wickham, 2023), ggplot2 (Wickham, 2016), rvest (Wickham, 2022), NADA (Lee, 2020), tidyr (Wickham and Girlich, 2022), ggbreak (Shuangbin et al., 2021), stringr (Wickham, 2019). All data and code are shared openly with this manuscript, ensuring the entire data analysis workflow is reproducible.

## 3. Results and Discussion

### 3.1. Abundance

**Fig. 3.**
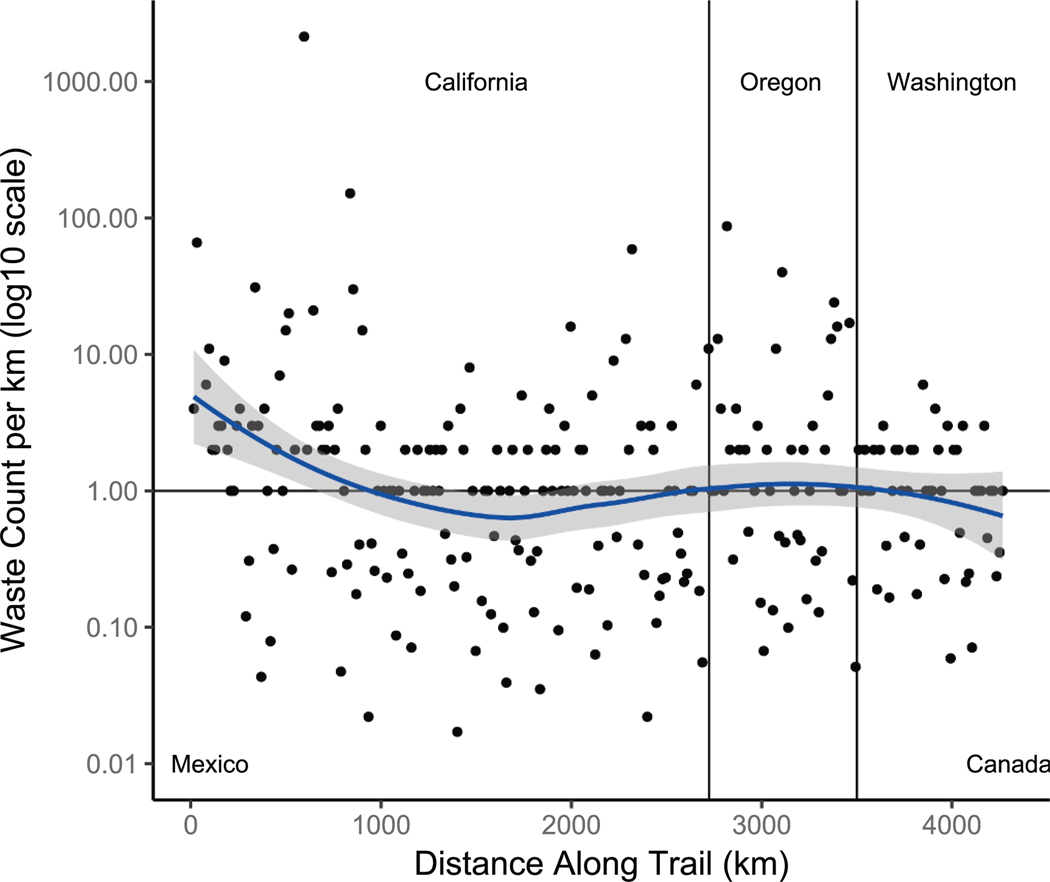
Counts observed along the trail with trail location on the x axis and waste abundance on the y axis. Blue smooth line with gray confidence intervals shows a generalized additive model with a smoothing spline to give an idea of the rough moving average. The center flat black line is the 1 count line; all data points below the line are extrapolated using cenros nondetect techniques.

Waste counts at the start of the trail in Southern California were relatively high, decreasing as hikers entered the Sierra Nevada mountains, then increasing closer to Oregon, and decreasing as hikers traveled further north into Washington (Fig. 3). Waste was found in 65 % of the surveys. Several large waste concentrations were apparent in the data. High waste sections of the trail were found to be related to the proximity of the trail section to roads, with less remote areas like Southern California and Oregon having a higher abundance of waste than the more remote Sierra Nevadas and Northern Washington (Fig. S2).

We estimated the total waste count on the trail to be 53,189 pieces (95 % confidence interval: 12,396–129,742) using the mean count per km of 12.5 (95 % confidence interval: 2.9–30.4) and extrapolating it to the entire trail. This average is similar to the 2.4 – 26.4 pieces/km range reported for macroplastics on trails in the Italian Alps (Parolini et al., 2021) and waste counts reported on trails in Maine and New Hampshire of 1.2–5.5 pieces/km (Wilkerson and Whitman, 2009). Given that the effects of resurveying and the number of surveyors suggested underreporting, this estimate is likely low by a factor of 2. However, for now, we feel more confident in the raw estimate and maintain that more research is needed to fully understand these biases before using them for extrapolation.

We compared the mean waste abundance on the Pacific Crest Trail (12.5 pieces per km) to concentrations in two other USA west coast systems studied by the authors using similar methodologies. A study in the Inland Empire of California found an average waste generation rate of 40,349 pieces per km per year (three orders of magnitude greater) on urban roadsides (Cowger et al., 2022), and a separate study found 2,697 pieces per km (two orders of magnitude greater) in urban river riparian areas in Pinole, California (Cowger et al., 2023). The Pacific Crest Trail was relatively pristine in comparison to either of these urban settings.

**Fig. 4.**
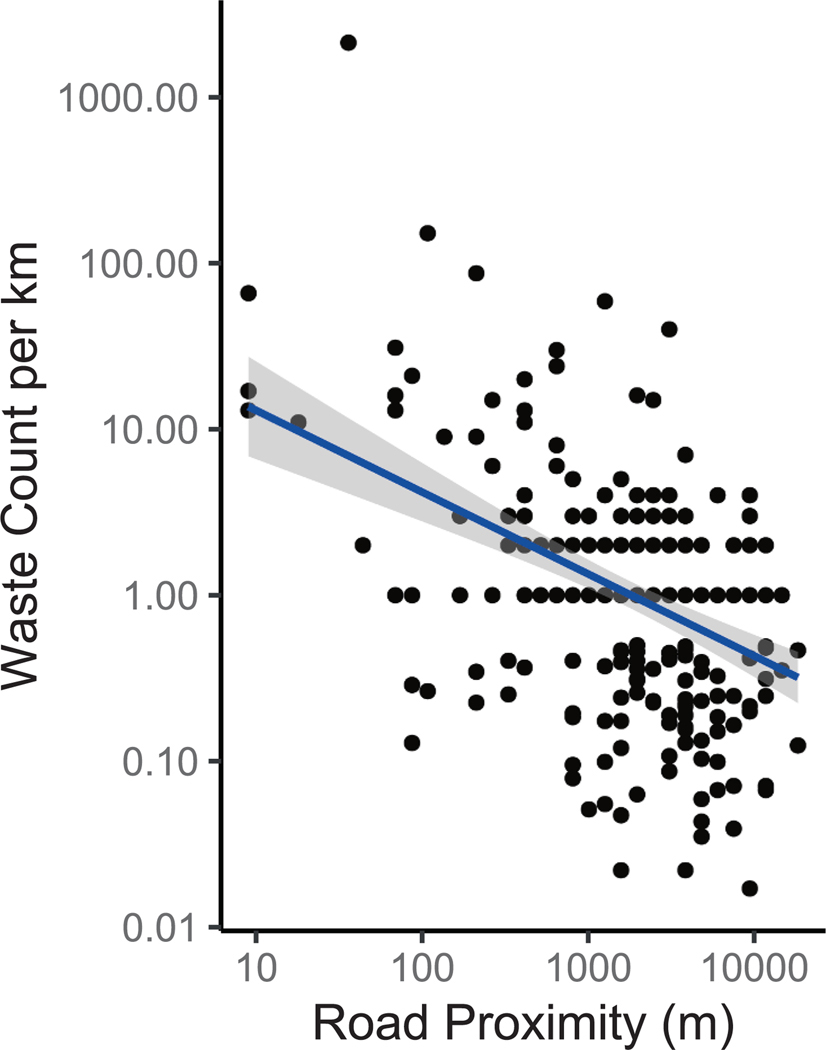
Observed waste counts per km (y axis) versus distance to nearest road (m) (x axis). Both axes are log_10_ scaled. A log–log linear regression (blue line) and 95% confidence interval (gray area) is shown.

A clear and expected relationship existed between distance to the nearest road and survey count (Fig. 4). The log–log linear regression statistics were log10(count) = −0.5 * log10(distance) + 2, slope p value = 10^−12^, y intercept p value = 10^−12^, adjRsquared = 0.2. This relationship could be used to predict a baseline of waste that regulators, land managers, and wilderness users can set a goal to achieve. We estimate the backcountry (10 km away from roads) has a count of 0.4 pieces per km (i.e., 50 % chance of finding one piece per 1 km). We used the same model to estimate that near-road (survey start 10 m from a road) stretches of the trail had 13 pieces per km. Future work could improve upon the model’s R^2^ of 0.2 (20 % of variance explained). It is likely that confounding factors, such as locations of waste receptacles, proximity to parking lots, trail popularity (e.g., average number of people on the trail each day), or information from hiking social networking applications (e. g., AllTrails) could explain some of the remaining variance.

### 3.2. Types

**Fig. 5.**
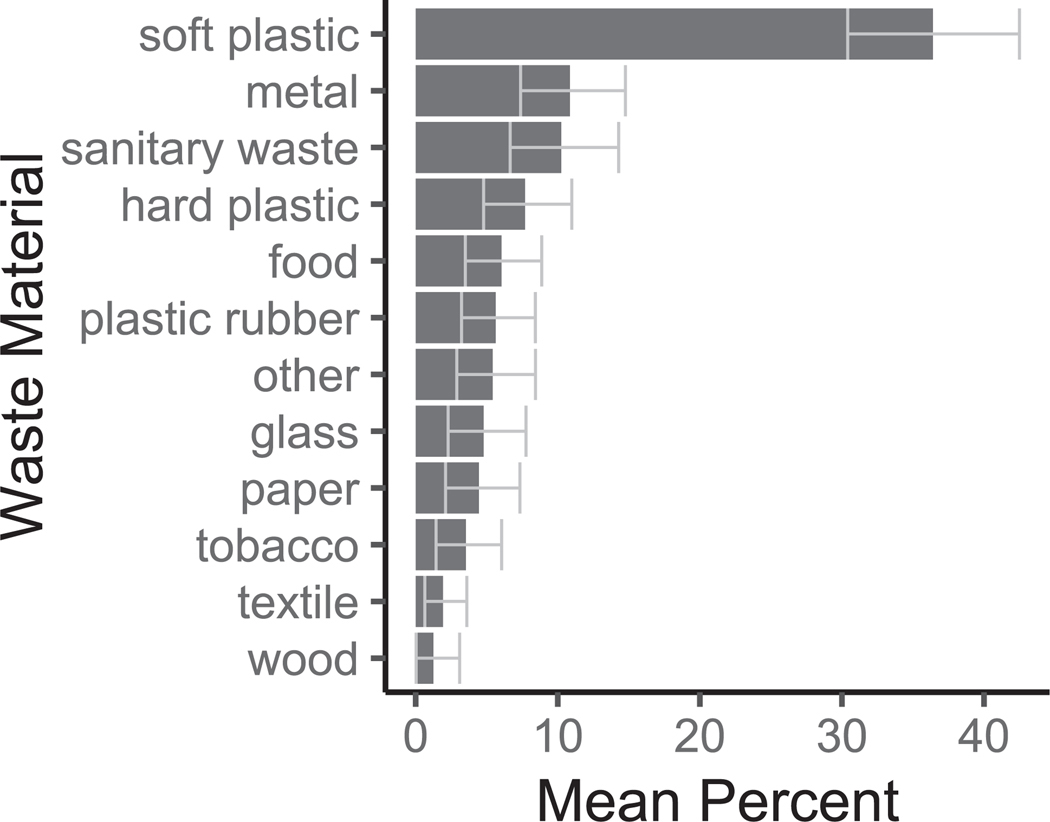
The top material types found on the trail. The mean percentage is plotted on the x-axis, and the y-axis represents the type of waste material. Bars represent the mean percentage, and whiskers indicate the 95% confidence intervals. Types are sorted from greatest (top) to least (bottom).

The most common waste materials were soft plastic (36 %), metal (11 %), sanitary waste (10 %), and hard plastic (8 %) (Fig. 5). Soft plastic is commonly ingested by animals, leading to intestinal issues (Ayala et al., 2023). We did observe soft plastic inside coyote scat (Fig. S 1) during one of the surveys, demonstrating that this impact may be relevant to the Pacific Crest Trail. However, this was only observed once, so its prevalence remains uncertain. Sanitary waste (e.g., soiled toilet paper) was the third most common waste material on the trail and presents biohazards to humans and animals. Textile pieces were one of the least common material types (2 %) observed on the trail, but microfibers are the most prevalent microplastic on trails (Forster et al., 2023). This suggests that microfibers in the environment may primarily come from textile shedding during use rather than fragmentation of large objects discarded on the trail. Food was the fifth-highest category (6 %), typically consisting of orange peels and sunflower seeds. These non-indigenous biodegradable materials are one of the lesser-known considerations for land stewardship. However, it is essential for normal ecosystem functioning to avoid introducing non-native biological materials into the wilderness (Marion et al., 2016). Leaving food waste encourages animals to interact with hikers and could introduce non-native species. Variation in waste materials across the trail did not follow a clear or consistent pattern (Fig. S 3).

**Fig. 6.**
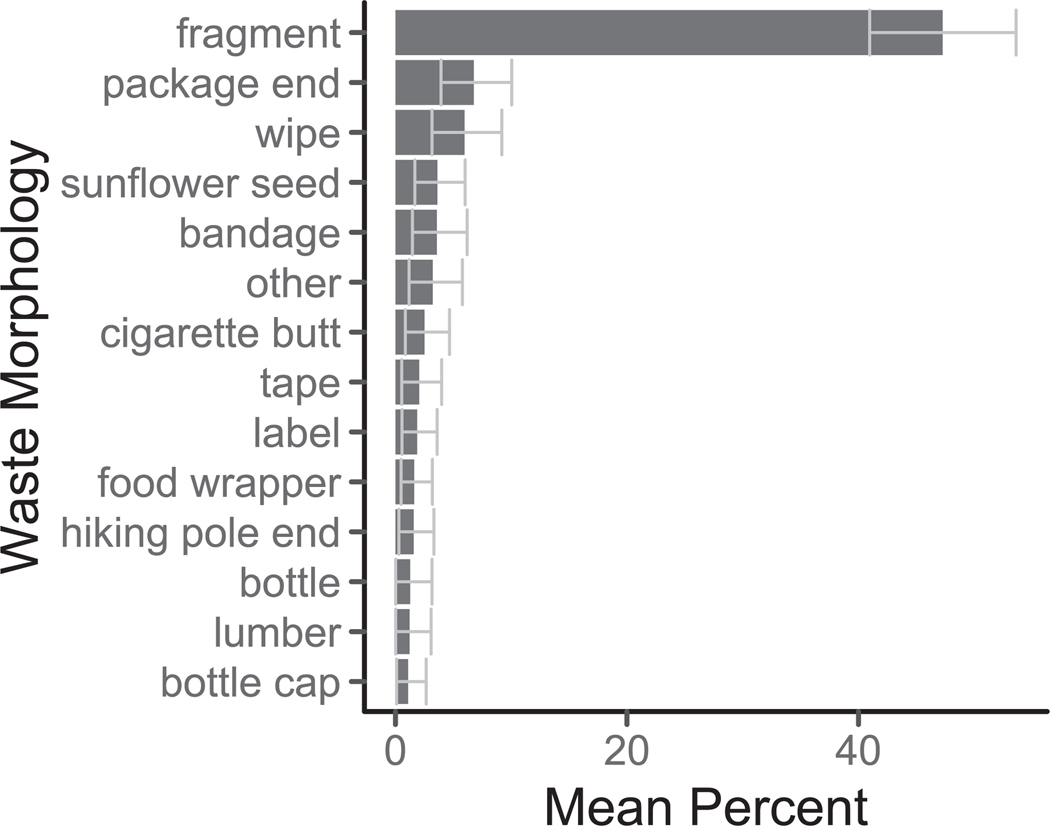
The top morphological types found on the trail. The mean percent is on the x-axis, and the y-axis is the waste morphology. Bars represent the mean percentage, and whiskers indicate the 95% confidence intervals. Only morphologies larger than 1% are shown. Types are sorted from greatest (top) to least (bottom).

Fragments (47 %), package ends (7 %), and wipes (6 %) were the most prevalent waste morphologies (Fig. 6). Fragments come from the breakdown of larger products (Julienne et al., 2019), are typically close to the particle size range of microplastics (< 5 mm), and are often more numerous by count but less abundant by mass (Kaandorp et al., 2023). Package ends come from the corners of rip-off tops of soft plastic packages meant for outdoor use (typically food packages). Generally, the prevalence of food-related morphologies (package ends, food wrappers, sunflower seeds, bottles, bottle caps) is unsurprising (Cowger et al., 2024b; Morales-Caselles et al., 2021; Parolini et al., 2021) as food is often consumed on the go, has a very short lifetime, and the packaging has a very low utility after the food is consumed compared to the other waste morphologies. Food packaging is common in other systems (Cowger et al., 2024b; Morales-Caselles et al., 2021) and has also been found in other trail waste studies (Parolini et al., 2021). Additionally, at the top of the list were morphologies not typically found in other ecosystems, such as outdoor gear list tape and hiking pole ends (2 % each) (Morales-Caselles et al., 2021). These products are used abundantly by hikers but are less likely to be used along roadsides or at beaches, for example. We collected data on visible brands on waste, but only found 48 pieces with identifiable brands. Fragments (the most prevalent type) do not typically have a brand on them. Future work could develop new methods for identifying producers of these fragments, as we are unaware of any suitable techniques, and these metrics would benefit companies tracking progress on their environmental and sustainability goals.

### 3.3. Method assessment

#### 3.3.1. Night effect

Occasionally, the surveyors reached a survey location after dark because there were insufficient camping locations prior to the survey. While this was rare, instead of skipping the survey, four surveys were conducted at night using headlamps to spot waste. The surveyors observed unique types of waste during these surveys, which were reflective under the direct headlamp light and waste that they thought may not be as visible under day conditions. The average count of waste found during daylight surveys was 12.3 (2.9–29.9), and the average during night surveys was 1.3 (0.1–4.0). We did not conduct a large enough assessment of these differences to be highly confident in the results, but this initial evidence suggests that night surveys may underestimate the waste count (potentially due to low ability to see the waste). However, correcting the night surveys using this relationship only changed the mean count per km estimate for all surveys from 12.5 to 12.6 because there were so few night surveys. Future work could explicitly be designed to assess if some waste types are more visible at night and by splitting the total number of surveys to have half of each type.

#### 3.3.2. Snow effect

Several survey locations (21 out of 251) were covered with snow during the survey. Instead of skipping these surveys, the effect of snow cover on the waste counts was evaluated. The mean waste count of snow-covered sites was 0.9 (0.5–––1.4), and the mean waste count of sites without snow was 13.6 (3.2–––33.6). Snow significantly affected the ability to observe waste, resulting in a 94 % reduction in recorded waste. This was expected because fresh snow would cover waste on the trail, and therefore, only very recent waste would be observable. Correcting the snow surveys using this relationship would change the mean waste count per km estimate from 12.5 to 13.5. Similar to the night effect, the relatively small overall impact on waste count per km is likely because few survey locations were snow-covered, and the adjustment would only apply to them. However, future studies may want to consider this effect in their survey design by avoiding snow-covered sites or conducting surveys during snowless seasons to standardize the assessment.

#### 3.3.3. Resurvey effect

Eight surveys were repeated to determine if the surveyors had missed any waste during their initial pass. The mean waste count of first-pass surveys was 6.5 (1–––13.7), while the repeat survey detected an additional 2.9 (0.5–––5.9) pieces on average. The 44 % underestimation of the waste count from the first-pass survey alone was large, suggesting that additional repeat surveys could have improved accuracy. If we corrected all surveys using this relationship to predict the waste counts if all surveys were repeated, the mean count per km estimate would increase from 12.5 to 28.4, suggesting this effect could greatly impact true counts. California waste monitoring surveys in rivers have been demonstrated to have a similar magnitude of bias (Moore et al., 2021). Future studies could improve on this method by increasing the number of repeat surveys and further validating this effect. While we repeated approximately 3 % of our surveys, increasing the number of repeated surveys to 10 % of all surveys, as time and resources allow, would provide a more accurate estimate.

#### 3.3.4. Number of surveyors effect

**Fig. 7.**
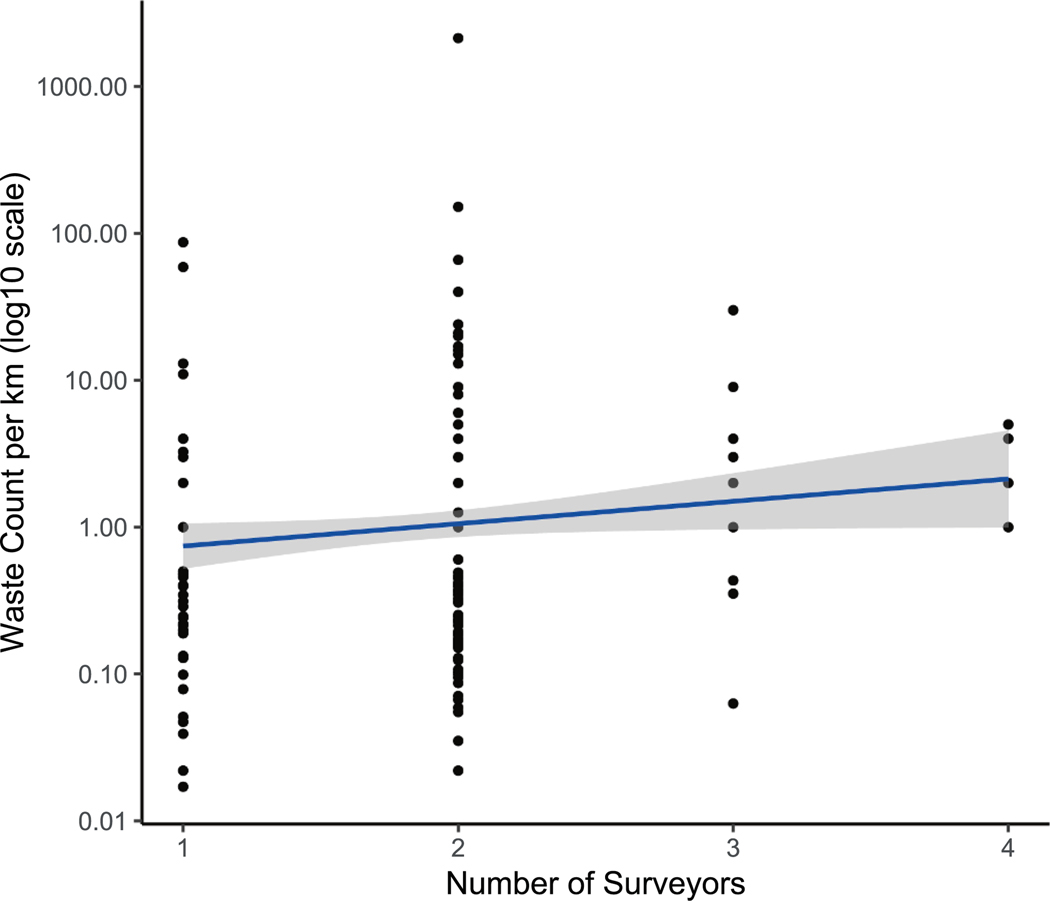
Observed waste counts per km (y-axis log scaled) versus number of people involved in the survey (x-axis), with log-linear regression (blue line) and 95% confidence interval (gray area).

The number of surveyors ranged from 1 to 4, with 59 surveys involving 1 person, 163 surveys involving 2 people, 16 surveys involving 3 people, and 4 surveys involving 4 people. The mean number of people was 1.9. A log-linear regression analysis was performed between the log_10_-transformed waste count and the raw number of surveyors (Fig. 7). The regression statistics were log10(count) = 0.2(surveyors) −0.3, with a slope p-value of 0.04, an intercept p-value of 0.06, and an adjusted R-squared of 0.01. If we corrected all surveys using this relationship to estimate the waste counts that would have been observed if 4 people had conducted all surveys, it would change the mean waste count per km estimate from 12.5 to 25.8. This factor of two increase is likely because two surveyors conducted most surveys. The resurvey effect and the number of surveyors effect were similar in size and impact, suggesting that resurveying has a similar effect to doubling the number of people, adding further evidence to support the validity of the observed effects. Future surveys could improve on the methods of this study by having a larger team (> 4) conduct all surveys.

### 3.4. Management recommendations

Based on the results found in this study, we propose three priority actions to reduce waste on the trail: cleanup, reengineering of materials, and adjusting waste management targets. To make the best progress on any of these recommendations, all pillars of society (government, community, and business) should work together. Road proximity strongly correlated to waste abundance, suggesting that targeted cleanups near roads, e.g., at trailheads, would greatly reduce waste on the trail. Wilderness waste management education for hikers appears to be keeping waste at low levels, relative to near-road conditions, even though many people hike the Pacific Crest Trail each year. The practices of hikers cleaning up after themselves and others should continue (Hu et al., 2018).

Manufacturers of goods used outdoors also have a role in helping address waste. Plastics were prevalent and are known to be persistent in the environment; there are likely better alternatives. Many outdoor food products come in individually wrapped packages, which could be replaced by bulk goods and reusable packaging wherever possible (Bergmann et al., 2023). Package ends were also prevalent, indicating a design flaw, as packages with serrated rippable ends are made to be broken into many small pieces easily. Packages that are designed to remain intact would be better to avoid environmental loss. Hiking pole tips and clothing tags often have a similar issue; they frequently fall off during use and could be better secured to the product. Wet wipes used for hygiene were prevalent on the trail. These products are often marketed as “flushable” which gives the perception that they are degradable or environmentally friendly, but they are neither (Allison et al., 2023). Manufacturers must rectify this inaccurate messaging and enhance their marketing efforts to promote the responsible use and management of their products (e.g., “wipes should be packed out of the wilderness and disposed of in a waste receptacle”). The composition of waste detected on the Pacific Crest Trail reflects the composition of products people use in the wilderness; therefore, producers should consider the toxicity, persistence, and health implications of products marketed for outdoor use.

Road waste management in California has established a threshold for acceptable conditions of 100 pieces per km (CalTrans 2023). A road waste threshold higher than the current near-road wilderness baseline estimates (13 pieces per km) could lead to increased wilderness impacts from road waste. This finding suggests that the current California thresholds should be at least one order of magnitude lower to maintain near-road wilderness conditions. Ideally, the long term goal would be to achieve the backcountry baseline of 0.4 pieces per km along all roadsides. We fully acknowledge that achieving a wilderness target on a road is a lofty goal and one that will not come without considerable effort and time. However, perhaps there are already roads out there that meet this threshold, and they should be modeled after and celebrated.

## 4. Conclusions

We estimate that there were 53,189 pieces of waste on the Pacific Crest Trail in 2023. The strongest predictor of trail waste abundance was proximity to nearest road with trails close to roads having the highest waste abundance. The most prevalent waste types were soft plastic and fragments. Assessment of this new method demonstrated that it underestimated waste abundance, with resurveys and doubling the number of surveyors increasing observed counts by 100 %. Night surveys and surveying snow-covered areas also considerably reduced observed counts in those locations, but few surveys were conducted under such conditions, so they did not have a significant impact on the research findings. All pillars of society, including government, community, and business, should collaborate to address trail waste, beginning with targeted cleanups, developing new products with reduced environmental footprints, and advocating for ambitious waste management goals.

## Supplementary Material

Supplemental Material McGruer et al. (2025)

## Data Availability

All data and code are available on Zenodo at: https://doi.org/10.5281/zenodo.15002831

## Appendix A. Supplementary data

Supplementary data to this article can be found online at https://doi.org/10.1016/j.wasman.2025.115063.
