# Supplementary material for: Lost on the Pacific Crest Trail: a 4,270 km survey of wilderness waste distribution and characteristics
Source: Waste Manag. Author manuscript; Available in PMC 2026 Mar 4. (PMC12959263; doi:10.1016/j.wasman.2025.115063)
Supplement: Supplemental Material McGruer et al. (2025) [file NIHMS2140201-supplement-Supplemental_Material_McGruer_et_al___2025_.docx]

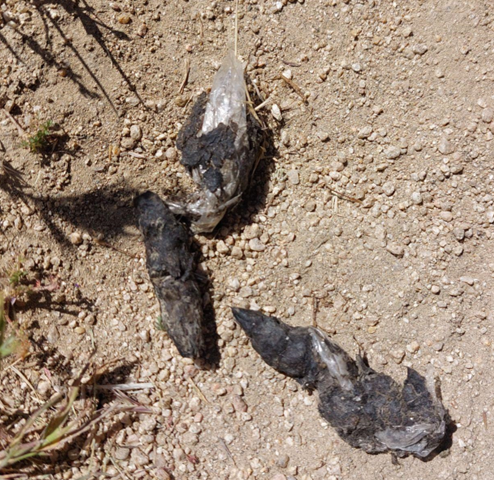


Figure S1: Soft plastic in a suspected coyote scat found on the trail during one of the surveys.


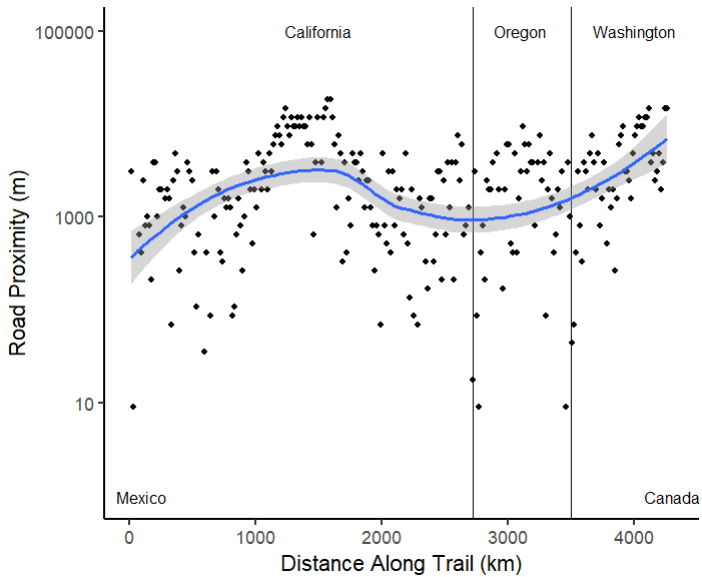

Figure S2: Relationship between road proximity (log10 scaled) and length of trail. Figure shows inverse relationship of Figure 2 in main text.


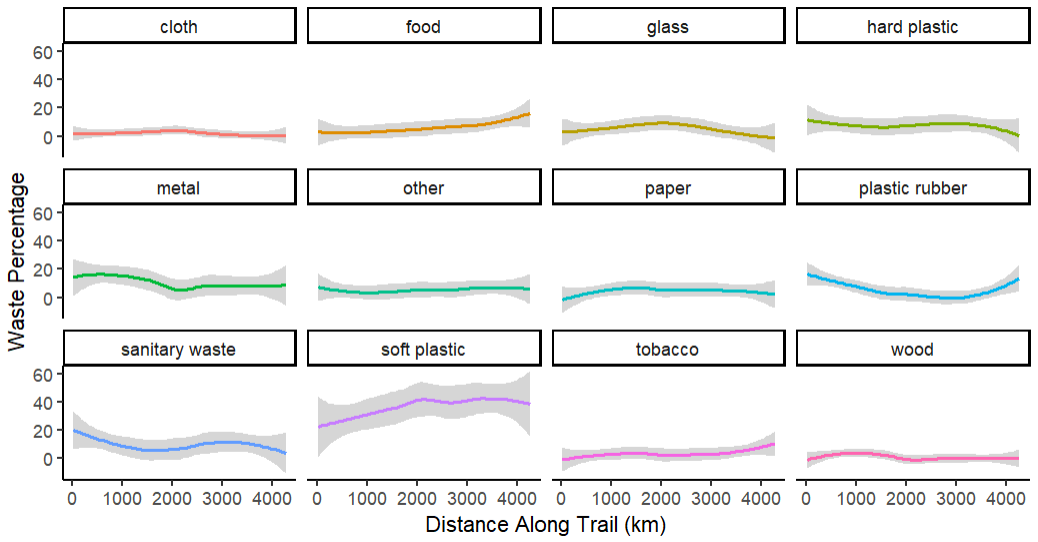


Figure S3: Analysis of waste materials across the trail. Centerline is the smoothing spline generalized additive model, the gray region is the 95% confidence interval.

## Trail Survey Methodology

1. Safety first, if a survey site is unsafe to survey for any reason, skip it.
2. Maintain trail awareness. It can be easy to turn down the wrong trail when you are focused on the survey. Look up often and make sure you are heading the right way!
3. Begin the trash survey by taking a photo looking up the trail - tag this photo as "survey start".
4. Record the start time of the survey.
5. Record how many people are conducting the survey.
6. Walk the trail at a constant pace observing trash on both sides of the trail and on the trail out to 2 meters on each side of the trail (approximately the distance made with both arms stretched out and hiking poles).
7. When trash is encountered, photograph it in place using one photograph with the Rubbish app (iPhone) or with a cell phone camera (Android)(Figure 8).


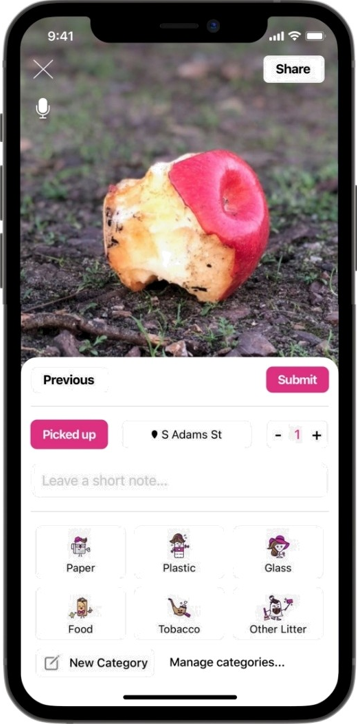


Figure 8: Example of Rubbish app interface for data collection.

- 1. Take the photo as soon as you find the trash (Figure 9).
  2. Take only one photo of each object.
  3. Do not record trash outside of the survey area.
  4. Take the photo within 1 m of where the trash lays.
  5. It is best to take photos of a single object at a time but it is sometimes necessary to take a photo of multiple objects simultaneously e.g., one item is broken into many. If photographing multiple objects, place only similar things in an image together.
  6. Photos should be close up but you should be able to see the entire item with enough background behind it to tell if it is against a fence, on the trail in a bush etc.
  7. Any brands on the objects should be visible in the image.
  8. If there is a pile of trash, deconstruct the pile and take photos of alike objects together in one image or photograph each piece individually.
  9. If there is a bag full of more than 10 items of trash, just photograph the bag.
  10. If there are items that cannot be removed because they are hazardous or too large, just photograph them.
  11. Open up any receipts so that the entire receipt can be read from the image.
  12. Photograph anything the size of a cigarette butt (approximately 1 cm) and larger.
  13. Retake any blurry photos or photos that do not have the entire object in view.
  14. Trail magic (items left for hikers) will be included as trash if it has no further foreseeable use (e.g., an empty crushed water bottle labeled "Trail Magic".)
  15. Camping equipment will be counted as trash if it appears abandoned.
  16. If photos of the trash cannot be collected due to the hazards of collecting it (e.g., on the edge of a cliff), then it will be recorded in the field notebook.


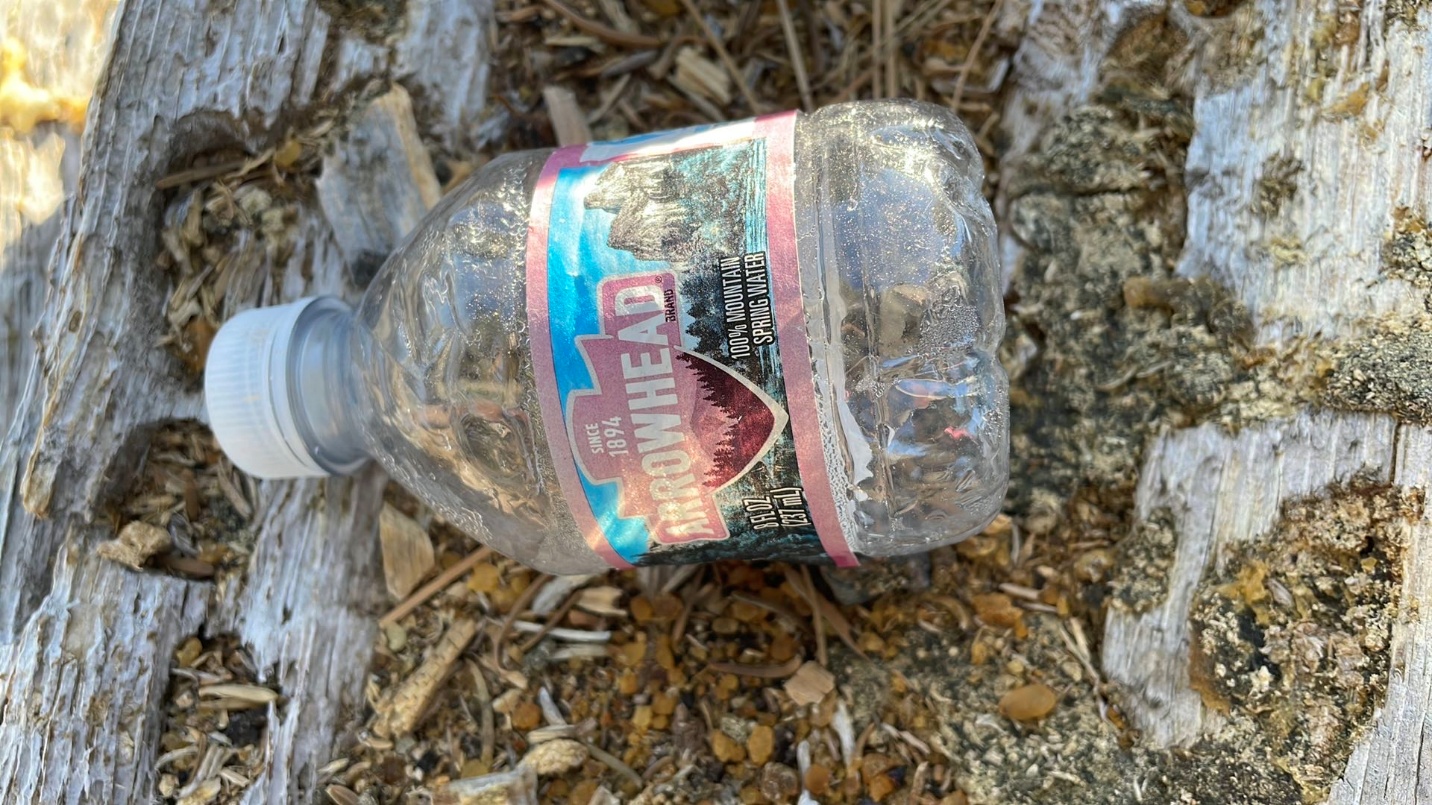


Figure 9: Example of a good-quality photograph with object and brand clearly visible.

1. Describe the trash by material, morphology, and brand type in the Rubbish or Android app.
   1. Use the words from the trash terms list for materials and morphology identification and copy the name exactly, spaces, and all.
   2. Materials are what the object is physically made of (e.g., soft plastic in Figure 9), while morphology describes its shape (e.g., food wrapper in Figure 9), and the brand defines the company logo (e.g., Smarties in Figure 9).
   3. If a material or morphology is not on the list, you can put it into an "other" category or create a new one and use that term each time you encounter that object.
   4. If the object is a mix of many materials, use the mixture's most abundant (by volume) material.
   5. If there are many objects, tag them all.
   6. Add the brand name as a tag if there is one or leave it blank.
2. Pick up the trash if possible. Large trash (e.g., a couch), hazardous trash (e.g., gasoline), or trash that could be interpreted as a historic artifact (very old > 50 years) will be left in place.
3. Track the total distance walked using GPS until you reach 1 km total survey distance.
4. Log the time the survey is completed.
5. Take a photo of the trail facing the completed section - tag this photo as "survey end".
6. Several sites should be surveyed a second time to estimate how much trash may be missed on a single-pass survey.
7. If 100 pieces of trash are encountered before reaching the end, record the last piece's location and end the survey there.
8. If the survey is a resurvey for quality control, label it in the field notebook.
9. What should you do with the trash?
   1. Reuse it: if you find a $100 bill, that is yours, buy yourself something nice.
   2. Upcycle it: make an art piece.
   3. Recycle it: check your local guidance on what can be recycled and how.
   4. Waste it: if none of the above is possible, throw it in your trash bin or take it to the local landfill.
